# “I’ll tell you what’s important to me…”: lessons for women’s health screening

**DOI:** 10.1186/s12905-021-01220-9

**Published:** 2021-02-18

**Authors:** Bayla M. M. Ostrach

**Affiliations:** Department of Research, UNC Health Sciences at MAHEC; Family Medicine and Medical Anthropology, Boston University School of Medicine, 121 Hendersonville Rd., Asheville, NC 28803 USA

**Keywords:** Screening, Women’s health, Human-centered design, Community engagement, Qualitative interviews, Appalachia, United States

## Abstract

**Background:**

Providers face increasing demands to screen for various health issues. Family medicine, primary care, and obstetric providers are encouraged to screen women universally for intimate partner violence, which could be challenging without comprehensive screening tools. The screening expectations and demands motivated providers and staff in south-central Appalachia (U.S.) to engage community members in streamlining women’s health screening tools, and integrating intimate partner violence screening questions, through a Human-Centered Design (HCD) process. The objective of this article is to present participants’ experiences with and perceptions of the HCD process for developing screening tools for women’s health.

**Methods:**

This was a qualitative, phenomenological study conducted with community members (*n* = 4) and providers and staff (n = 7) who participated in the HCD process. Sampling was purposive and opportunistic. An experienced qualitative researcher conducted open-ended, semi-structured interviews with participants. Interviews were transcribed and coded for thematic analysis.

**Results:**

Community members reported that in the HCD sessions they wanted clinicians to understand the importance of timing and trust in health screening. They focused on the importance of taking time to build trust before asking about intimate partner violence; not over-focusing on body weight as this can preclude trust and disclosure of other issues; and understanding the role of historical oppression and racial discrimination in contributing to healthcare mistrust. Providers and staff reported that they recognized the importance of these concerns during the HCD process.

**Conclusions:**

Community members provided critical feedback for designing appropriate tools for screening for women’s health. The findings suggest that co-designing screening tools for use in clinical settings can facilitate communication of core values. How, when, and how often screening questions are asked are as important as what is asked—especially as related to intimate partner violence and weight.

## Background

Clinicians are keenly aware of increasing pressures and expectations to implement various screening tools in primary care and women’s health. Demands on clinician time for screening have long been recognized. A 2003 estimate predicted it would take up to 7 h a day to comply with all of the U.S. Preventive Services Task Force recommendations [1]. Since then, the number of recommendations has only increased. Meanwhile, public health experts, medical anthropologists, and increasingly, providers, focus on areas where screening needs to be more nuanced: to reflect preconception and interconception health needs, and to take into account social determinants and structural factors that predict racialized and structurally produced disparities in reproductive outcomes [2–5].

Human-Centered Design is an innovation and design process that emphasizes collective input from stakeholders [6–8]. An increasingly popular framework for identifying a problem and moving quickly to create a product or determine a course of action, HCD was initially used in business, marketing, and consumer design [6, 8, 9]. The use of HCD rapidly expanded into computer science [10], visual design [11], and healthcare [7]. A growing body of literature describes healthcare products and processes resulting from such patient engagement [12] and examines its utility for improving provider-patient communication and treatment plan development, visit attendance, and satisfaction [6, 8, 9]. It thus represents one possible tool for provider and patient/community engagement. Yet the extant literature on HCD does not document or explore participant experiences with and perceptions of, being involved in HCD processes nor the lessons to be learned from participants’ experiences in HCD processes. This study contributes to filling this gap in the literature.

The tension between screening expectations and demands on provider time, as well as an awareness of the need to reflect structural conditions that affect reproductive health outcomes [4, 5] motivated a group of clinicians at a primary care health center in the Southeast United States (U.S.) to undertake a HCD process in order to redesign and develop new screening tools for women’s^1^ health issues. These included: pregnancy intention and contraception, multivitamin with folate use, body weight, physical activity, tobacco, alcohol, and substance use, depression, intimate partner violence, and sexual activity. The goal was to combine these questions into one streamlined tool [13]. Community members were integral to the process of developing the screening tools [13, 14]. The specific HCD process involved engaging key stakeholders from local communities historically under-represented in health screening design [13, 14] and clinicians with some experience working with these populations, particularly in primary care settings. The process was planned around creating comprehensive women’s health screening tools by synthesizing existing tools already being used in a network of family medicine clinics, and incorporating intimate partner violence (IPV) screening. Two HCD sessions focused on each goal, a third session was dedicated to testing a prototype screening tool developed in and from the earlier sessions [13].This article presents findings from a study that explored participants’ experiences with and perceptions of that HCD process.

While the study was designed to explore participant experiences with and perceptions of being involved in an HCD process to design a screening tool for women’s health and IPV, larger lessons from screening emerged: highlighting the perspectives of key actors involved in healthcare provision and community members regarding screening. Thus this study presents findings about more than an HCD process used for women’s health screening tool development.

## Methods

### Design

Qualitative approaches can shed important light on improving screening approaches and guidelines, as they capture nuances of patient experience and context that may otherwise be lost [15]. In this non-experimental qualitative study, the research question was intended to facilitate an understanding of participants’ experiences of a human-centered design process used to redesign women’s health screening tools.

### Ethical considerations

The study protocol was reviewed and determined not human subjects research by the relevant Institutional Review Board that reviews all studies for the medical education center that hosted this project. The author is not allowed to name the specific institutional review board/ethics committee that made this determination as it was, at the time of the study, the only such ethics committee that reviewed all studies for any research conducted at the study site: it is based at a hospital where all study participants either provide or receive care and connected to the network of family medicine clinics for which the screening tools were developed, thus naming the ethics committee would risk identifying the site and participants. Under the study protocol and consent process reviewed by the ethics committee, both the site and the participants must be kept de-identified. Naming the specific ethics review committee would violate the approved study protocol. All participants gave verbal informed consent. There were no incentives for participation in the study. The study did not gather participant-identifying information aside from connection to and role with the study site.

### Sampling and site

As a qualitative, phenomenological study, the sampling strategy was purposive [16–18], with participants recruited specifically because they had a shared experience of the HCD process.. Recruitment and data collection occurred through a linked medical education center and network of family medicine primary care clinics within a south-central Appalachian community [19].

### Recruitment and participation

Participants in the study included community members involved in the HCD process to redesign the screening tools as well as health center providers and staff who participated in the process and had direct involvement in patient care or supervised/trained those who do. All seven providers and staff who met inclusion criteria agreed to be interviewed and chose to do so at their place of work. Six community members met inclusion criteria; four agreed to participate and chose to be interviewed at the medical education center or at the community resource center of a local public housing facility. All four eligible community members who agreed to participate in the study were African-American women and had participated in all three HCD sessions.. Two community members who declined to be interviewed were Latinx women and had participated in only the second of the three HCD sessions. In all, a total of 11 of 13 eligible participants were interviewed.

### Data collection and analysis

The author, an experienced qualitative researcher, conducted all interviews using a semi-structured interview guide developed based on documentation of the HCD process itself, a literature review, and initial discussions with the medical education center staff who initiated the HCD process. Interviews were audio-recorded with the permission of participants. To conduct the interviews, the author used a semi-structured interview guide that included questions about: participant’s role (community member; patient; provider; staff), time in that role (if relevant), their decision to participate in the HCD process, what the experience was like for them, what they expected from it and their comfort level, their perceptions of what they heard about IPV screening and about women’s health screening, what was hard and/or easy about it, what they learned from the process, and anything else they wanted to share. All interviews were transcribed verbatim by an outside firm. The author conducted thematic analysis of verbatim transcripts [20], using a codebook developed based on a review of literature about HCD and health screening; initial informational conversations with the subset of providers and staff who initially sought to use the HCD process to redesign screening tools; and an iterative review of the first three transcripts. Salient codes from the three transcripts were identified, hand-coded, and used to thematically analyze all transcripts in order to identify important emerging themes, relationships between the themes, and the patterns across interviews [20]. Quotes from interviews included below to illustrate key themes have been edited for clarity while maintaining each speaker’s style of speech and emphasis.

## Results

In all, eleven providers, staff, and community members from the earlier HCD process participated in in-depth, open-ended interviews. Overall, all participating community members involved in the women’s health screening tool redesign process reported three ideas they had wanted health providers and staff to hear: (1) that screening for IPV requires spending time and earning trust; (2) a person’s weight should not be the focus of every medical visit and that making it the focus can create a barrier to earning trust; and (3) that their (African-American) community’s prior history of discrimination and distrust in medical settings interferes with effective health screening.

### Intimate partner violence screening

Each community member interviewed reported emphasizing the difficulty of expecting that someone would disclose past or current experiences of IPV during an initial screening at a medical appointment with a provider they might not know, or know well yet. Community members reported that during the HCD sessions, they wanted clinicians to understand that effective screening for IPV requires trust. They reported that they emphasized in the HCD sessions that trust requires more time, and thus more visits with a given provider for IPV screening to be effective. The desire to develop trust before disclosing experiences of IPV was not due to fear of increased risk of violence from a current partner, as it also extended to disclosure of past experiences and not just the situation at the time of the visit. The desire for trust to be gained over time and over multiple visits was described by community member participants purely in terms of not wanting to talk with providers and staff about something as personal, and literally, *intimate* as intimate partner violence in a setting that they perceived as a clinician simply checking a box to complete a required screening. The community member participants strongly felt that providers should not ask screening questions unless they can take time to discuss the answers; and that they should not “just check off boxes” for the sake of completing a screening if the information gained cannot be effectively used. The following quote from a staff member who took note of the message during the session exemplifies this view:

*Community members said, ‘*at that first visit, don’t ask me about intimate partner violence*…’ They kept saying, ‘*don’t – please don’t bring it up because I probably won’t tell you, but, I might tell you the next time I see you*…’ I kept hearing there has to be a trusting relationship built. Asking the questions needs to be at the right time… It’s not just a ‘check these boxes on this sheet.’ It’s a, ‘*move this out of the way*’* [pantomimes moving a laptop out of the way]*. ‘*Talk to me about it and I might go deeper with you…’ *Ask in the right way at the right time.*—Marie, practice manager

Likewise, SuzieQ,^2^ a community member who was an IPV survivor and at the time the only patient of the clinics for which the screening tools were being developed, stated:

*I think [clinicians] learned the questions not to ask… the questions they thought were helpful, they realize they’re not. They actually make you withhold more information. I think they paid attention to women needing space when they’re asking those questions. Don’t ask in front of [a] partner! [I told the clinicians] I had that experience –a doctor asked me,* ‘was I being abused?’ *[when] my abuser was right there.*

SuzieQ felt it was important that providers and staff in the HCD sessions understand that screening for IPV too soon or in the wrong way could deter someone from disclosing even a current situation of abuse that she spoke in the session about her own direct experience of exactly that, even though it was, as she said, “a delicate topic.” Though SuzieQ spoke more directly than did any other community member about her own past experiences with IPV, other community members alluded to sharing this experience, and all agreed that screening for IPV too soon could prevent someone from disclosing.

### Weight screening

Similar to talking about the need to gain trust in clinical settings in order to effectively screen for IPV, all community members interviewed wanted clinicians to understand that weighing them at the beginning of every visit, or discussing their body weight to the exclusion of other health issues as a main focus of every visit, interferes with trust. Community members reported that what they perceived as clinical hyperfocus on body weight shuts down communication about specific symptoms or health topics they may need or want to discuss during a medical visit. Every community member interviewed stated some variation of, “weight - don’t ask about it!” Community members did not specify that they would prefer less frequent weight screening, or that such screening should be conducted at a different time during an appointment; rather, they almost uniformly stated a preference that providers not treat them as though the number on the scale were a proxy for them as a person—seeing only their weight or Body Mass Index (BMI) instead of asking why they had come in to the clinic or what they wanted to talk about.

This perspective of community members regarding screening for weight was one that nearly all staff and providers reported in their interviews. Staff and providers interviewed frequently mentioned community members’ recommendation that less time and focus during medical visits be devoted to weight. As Raleigh, a clinic project manager who worked most closely with several of the community members on community maternal health programs, stated:

*Women started talking about their weight and how they wish their provider would* stop *talking about their weight. [They said] ‘*that’s the first thing [providers] talk about whenever they walk into the office…*’ that was a huge thing, ‘*you know, I don’t want to go into a doctor’s office because every single time [they bring it up and] I’m tired of talking about my weight…

### Historical and community mistrust

Community members interviewed, all of whom were African-American, narrated their own or family members’ past negative healthcare experiences that lead to current mistrust of medical providers and settings. They also talked about discrimination in healthcare settings and mistrust in their wider community based on historical legacies of medical racism and medical abuses of African-American people in the United States. Community members explicitly mentioned family and personal experiences, and historical events such as the Tuskegee Syphilis Study, as reasons that health screening would be challenging and why it needed to be designed and undertaken thoughtfully, with attention to local contexts. All community members recognized the role of racial inequality and historical oppression in medical mistrust and prevailing wariness about health screening. Some community members mentioned recent-generation family members or friends forcibly sterilized by public health doctors, including locally; and their own experiences with unwanted procedures during labor and delivery. These experiences shared during HCD sessions made lasting impressions on providers, as recounted by Zora, a staff person involved in organizing the screening tool redesign:

*A community partner felt dismissed [in] interactions with front desk staff [and] on the phone. We heard stories about Tuskegee, and forced sterilization. There were some personal stories. One woman talked about her grandmother [who] died because she wasn’t given the care she was due… it was generational. Her grandmother, her mother, and her, they all had really negative experiences.*

Community members also cited simpler aspects of medical visits that hindered trust: for example, providers looking at a computer screen or tablet while asking screening questions, rather than at the patient. Some participants also mentioned that how screening questions are worded can be offensive, particularly in relation not just to IPV but other sensitive topics such as pregnancy, contraception, substance use, and depression. Most community member participants mentioned that how screening is administered—the words used, and the style in which it is delivered, and the impact of both on trust, determines how effective it is. This view was noted by providers and staff:

*I would say the biggest take-away [I heard] is what clinicians try to achieve [with] screening is not as important as relationships [with patients]and how they feel valued… this screening may not be achieving the results we want because patients do not trust us.*—Dr. Why, Family Medicine provider

## Discussion

Clinical, public health, and social science experts endeavor to apply research findings to inform more nuanced approaches to the ever-increasing demand for primary care and women’s health screening—including seeking best ways to reflect social and structural determinants that may contribute to reproductive and sexual health and healthcare inequities [1, 2]. Yet even well-intentioned efforts to improve screening at times default to quantity over quality; community members in this study recounted perceptions of and preferences for women’s health screening that suggested their previous experiences with screening had been less than positive.

Though primary care providers face increasing expectations, backed by evidence-based public health guidance, to incorporate IPV screening into routine medical visits, this is usually not effective from the perspective of patients. Community members reflecting on the HCD process emphasized the risks of screening for IPV too soon before trust is established between a provider and patient. One community member even referenced her own negative experience of a provider asking her about IPV in front of an abusive partner to emphasize how a wrong approach to IPV screening could deter disclosure. This was a common perspective among community members; one of which some providers and staff took note. Yet the ability or willingness of the providers and staff involved in the HCD session to determine not only how, but when and how often IPV screening would occur, was unclear. Literature on women’s health and violence prevention, the U.S. Preventive Services Task Force guidelines, and primary care models of IPV screening all encourage universal IPV screening in primary care and women’s health. However, the findings of this study indicate that it is not just a question of where, by whom, and how often screening for IPV should occur, but also that when (at what visit) it should happen is of paramount importance. The findings strongly suggest a need to wait until rapport and a relationship are established between patients and their provider, prior to routine IPV screening.

Similar to IPV screening, community members expressed concerns about over-emphasis on weight during clinic visits. Community members described how they, uniformly, told the providers and staff during HCD sessions that screening for weight at the beginning of every medical visit, and talking about weight during every visit (especially to the exclusion of other topics, or as the primary focus) can impede or prevent the establishment of a trusting relationship—and thus interfere with effective screening for other health issues. The perceptions of community members regarding providers’ over-emphasis on weight during clinic visits is indicative of the use of BMI as a health indicator that disproportionately labels non-white women at greater risk based on body size [16, 21, 22]. The findings of this study indicate that too frequent or over-emphasis on weight screening might even preclude effective IPV screening; with the latter already rendered less effective when done at a first visit. Although existing healthcare guidelines recommend weight screening as part of primary and women’s health care, the findings of this study suggest that the timing, location, and format of such screening has implications for how effectively other screening is received. The findings suggest effective screening procedures for weight require an understanding of patients’ preferences to ensure trust is not compromised.

Overall, community members described the interrelatedness and importance of past healthcare experiences, an awareness of medical racism and historical medical abuses, and trust informing any given medical encounter, and influencing screening effectiveness and impact. Community members reported that perceived racial/ethnic discrimination and past mistreatment create barriers to trust in healthcare settings, as do the manner and timing of screening. Providers and staff reported taking note of these concerns during HCD sessions. With increasing emphasis on social determinants of health and health equity in both medical education and screening guidelines [2–5], clinicians’ awareness of such perspectives raised in an HCD session could, ideally, inform not only a set of screening tools but also broader conversations in the clinical setting.

A process intended to redesign women’s health screening tools that brought together community members, specifically women of color in a historically underserved area, in conversations with clinicians resulted in the creation of streamlined, simplified screening products that were then used in all primary care sites affiliated with the regional medical education center and network of clinics where this study was conducted. At the time of this writing two materials developed through the HCD process were in use at the primary care clinics, for (perceived or assigned at birth) women’s first appointments; annual exam appointments; at set intervals during prenatal care; and at intervals since the most recent appointment (determined by providers, but not less often than 1 year). The materials are a graphically designed, artistically formatted ‘conversation starter’ brochure provided at check-in intended to give culturally relevant positive context for the importance of the selected screening topics; and a laminated, one-page screener with multiple choice check-off questions that match topics on the patient brochure: pregnancy intention and contraceptive use; satisfaction with body weight; physical activity levels; multivitamin and folic acid use; tobacco use; alcohol/drug use; depression scale; intimate partner violence; and sexual activity [13]. The screener is administered by a medical assistant or self-administered, with a follow-up conversation that occurs if a patient screens positive on certain topics. The follow-up conversation is intended to be centered on the patient’s priorities and desires. Both materials were designed with input from the HCD sessions, and reflect lessons learned in the process. Though the content and design of the tools reflect community members’ input from the HCD sessions, the way they are administered does not.

### Limitations

One of the limitations of this study is that participants were asked specifically about their experiences with and perspectives of an HCD process used to design women’s health screening tools, not about their views of health screening generally. While this may have limited the depth of the study’s findings about participants’ views of comprehensive health screening overall, this was not the focus of the study and was not the focus of what stakeholders sought to learn. Moreover, as the HCD sessions addressed IPV and screening for various health conditions, all participants did report on some aspects of screening more generally. Community members recruited for the HCD sessions were all women of color, their perspectives may therefore not be representative of others who might receive care at clinics intending to use the newly developed screening tools. However, this is also a strength, as these perspectives are rarely reflected in the development of health screening tools, or in qualitative research about primary care.

## Conclusions

Multiple conclusions can be drawn from the process of involving clinicians and community members in redesigning women’s health screening tools. First, when recognized as experts on their own care, community members convey far more than simple feedback about the wording, ideal number and timing of screening questions to be asked during clinic visits. Involving patients in co-designing tools for screening can facilitate communication of core values and priorities. Key messages from such involvement were that (1) intimate partner violence screening in primary care settings requires trust earned over time; (2) over-emphasis on weight screening and body size is a barrier to trust in medical settings and can be a deterrent to full disclosure; and (3) historical oppression and experiences of discrimination in healthcare lead to prevailing mistrust and wariness among patients. In essence, *how, when,* and *how often* screening questions are asked are as important as what is asked—particularly in relation to intimate partner violence and weight. The findings of this study suggest that repeated interactions, relationship-building, and increased trust are vital for ensuring effective health screening.

For clinicians and practice managers implementing health screening, the findings of this study suggest the importance of:

1. Planning IPV screening at intervals such that ample time is allowed for trust to be established between patients and providers—especially those directly administering screening, that may not be the physician, but instead a medical assistant or nurse;
2. De-emphasizing weight screening; and if recording weight is required for accreditation or quality metrics, considering ways to measure and record it without making it a major focus of the medical visit—for example by having the patient weigh themself at the end of the visit and record it on a piece of paper for the medical assistant to enter later, or give the option to be weighed at the end of the appointment without being told the result; and
3. Educating all providers and staff in the history, legacy, and prevailing landscape of medical racism and racialized health and healthcare inequities faced by many patients and how these likely affect access to care, and trust.

## Data Availability

Given the nature of the interview questions, the identifiability of the site, the characteristics of the study participants, and the stipulations of study protocol reviewed by the ethics committee, including the specific consent process which included participants receiving assurance that no-one other than transcriptionists and study team members would have access to transcripts of their interviews, the author is explicitly disallowed from making study data available in any form. To do so would violate the terms of the ethics review.

## Abbreviations

HCD: Human-centered design
IPV: Intimate partner violence

## Footnotes

1 Throughout this paper, “women” refers to cisgender women and/or patients assumed by their healthcare providers to be the sex and gender they were assigned at birth. While the author recognizes that transgender women and non-binary people assigned female at birth are as or more likely to experience comparatively more negative health outcomes, at the time the HCD process was undertaken the medical center staff leading it chose to focus on people assigned female at birth and perceived as women. Similarly, existing literature on women’s health screening largely does not differentiate or clarify whether “women” means ciswomen, or all women.

2 All names are pseudonyms chosen by participants.

## References

[1] Yarnall KSH, Pollak KI, Østbye T, Krause KM, Michener JL (2003). Primary care: is there enough time for prevention?. Am J Public Health.

[2] Frayne DJ, Verbiest S, Chelmow D, Clarke H, Dunlop A, Hosmer J (2016). Health care system measures to advance preconception wellness: consensus recommendations of the clinical workgroup of the National Preconception Health and Health Care Initiative. Obstet Gynecol.

[3] Kuzawa CW, Gravlee CC (2016). Beyond genetic race: biocultural insights into the causes of racial health disparities. New Dir Biocultural Anthropol.

[4] Ostrach B. Invited brief: quality reproductive care depends on understanding how inequality exacerbates multiple health problems. Scholars Strategy Network; 2017. http://www.scholarsstrategynetwork.org/sites/default/files/ssn-key-findings-ostrach-on-reproductive-health-syndemics.pdf.

[5] Wallace M, Crear-Perry J, Richardson L, Tarver M, Theall K (2017). Separate and unequal: structural racism and infant mortality in the US. Health Place.

[6] Burton B, Henry M, Mccolgin D (2012). The evolution of human-centered innovation: designing for empathy. Ethnogr Prax Ind Conf Proc.

[7] Matheson GO, Pacione C, Shultz RK, Klügl M (2015). Leveraging human-centered design in chronic disease prevention. Am J Prev Med.

[8] Harte R, Glynn L, Rodríguez-Molinero A, Baker PM, Scharf T, Quinlan LR (2017). A human-centered design methodology to enhance the usability, human factors, and user experience of connected health systems: a three-phase methodology. JMIR Hum Factors.

[9] Harte R, Quinlan LR, Glynn L, Rodríguez-Molinero A, Baker PM, Scharf T (2017). Human-centered design study: enhancing the usability of a mobile phone app in an integrated falls risk detection system for use by older adult users. JMIR MHealth UHealth.

[10] Kurosu M (2009). Human centered design: first international conference.

[11] Parsons P, Sedig K, Didandeh A, Khosravi A. Interactivity in visual analytics: use of conceptual frameworks to support human-centered design of a decision-support tool. In: 2015 48th Hawaii international conference on system sciences. 2015. p. 1138–47.

[12] Hartzler AL, Chaudhuri S, Fey BC, Flum DR, Lavallee D (2015). Integrating patient-reported outcomes into spine surgical care through visual dashboards: lessons learned from human-centered design. eGEMs.

[13] Foley KA, Shelton J, Richardson E, Smart N, Smart-MacMillan C, Mustakem O (2019). Primary care women’s health screening: a case study of a community engaged human centered design approach to enhancing the screening process. Matern Child Health J.

[14] Ostrach B (2020). Human-centered design for a women’s health screening tool: participant experiences. South Med J.

[15] Abadir AM, Lang A, Klein T, Abenhaim HA (2014). Influence of qualitative research on women’s health screening guidelines. Am J Obstet Gynecol.

[16] Rogge MM, Greenwald M, Golden A (2004). Obesity, stigma, and civilized oppression. Adv Nurs Sci.

[17] Charmaz K (2006). Constructing grounded theory: a practical guide through qualitative research.

[18] Bernard HR (2011). Research methods in anthropology: qualitative and quantitative approaches.

[19] Appalachian Regional Commission. Appalachian Subregions. 2002. http://www.arc.gov/images/maps/22512_Subregions.pdf.

[20] Creswell JW (2012). Qualitative inquiry and research design: choosing among five approaches.

[21] Byrne C. The BMI is racist and useless. Here’s how to measure health instead. HuffPost Life. Huffington Post. https://www.huffpost.com/entry/bmi-scale-racist-health_l_5f15a8a8c5b6d14c336a43b0?utm_campaign=share_twitter&ncid=engmodushpmg00000004.

[22] Moharram MA, Aitken-Buck HM, Reijers R, van Hout I, Williams MJ, Jones PP (2020). Correlation between epicardial adipose tissue and body mass index in New Zealand ethnic populations. N Z Med J.

